# Supplementary material for: Physicochemical Properties of Nanoencapsulated Essential Oils: Optimizing D-Limonene Preservation
Source: Polymers (Basel). 2025 Jan 27;17(3):348. doi: 10.3390/polym17030348 (PMC11820669; doi:10.3390/polym17030348)
Supplement: Supplementary file 1 [file polymers-17-00348-s001.zip › polymers-3259279-supplementary.pdf]

Supplementary Materials: Physicochemical Properties of Nanoencapsulated Essential Oils: Optimizing D-limonene Preservation

Diner Mori-Mestanza [1], Iraida Valqui-Rojas [1], Aline C. Caetano [2], Carlos Culqui-Arce [1], Rosita Cruz-Lacerna [1], Ilse S. Cayo-Colca [3], Efraín M. Castro-Alayo [1] and César R. Balcázar-Zumaeta [1,*]

[1] Instituto de Investigación, Innovación y Desarrollo para el Sector Agrario y Agroindustrial (IIDAA), Universidad Nacional Toribio Rodríguez de Mendoza de Amazonas, Chachapoyas 01001, Amazonas, Peru
[2] Instituto de Investigación para el Desarrollo Sustentable de Ceja de Selva, Universidad Nacional Toribio Rodríguez de Mendoza de Amazonas, Calle Universitaria N° 304, Chachapoyas 01001, Amazonas, Peru
[3] Facultad de Ingeniería Zootecnista, Agronegocios y Biotecnología, Universidad Nacional Toribio Rodríguez de Mendoza de Amazonas, Chachapoyas 01001, Peru
* Correspondence: cesar.balcazar@untrm.edu.pe

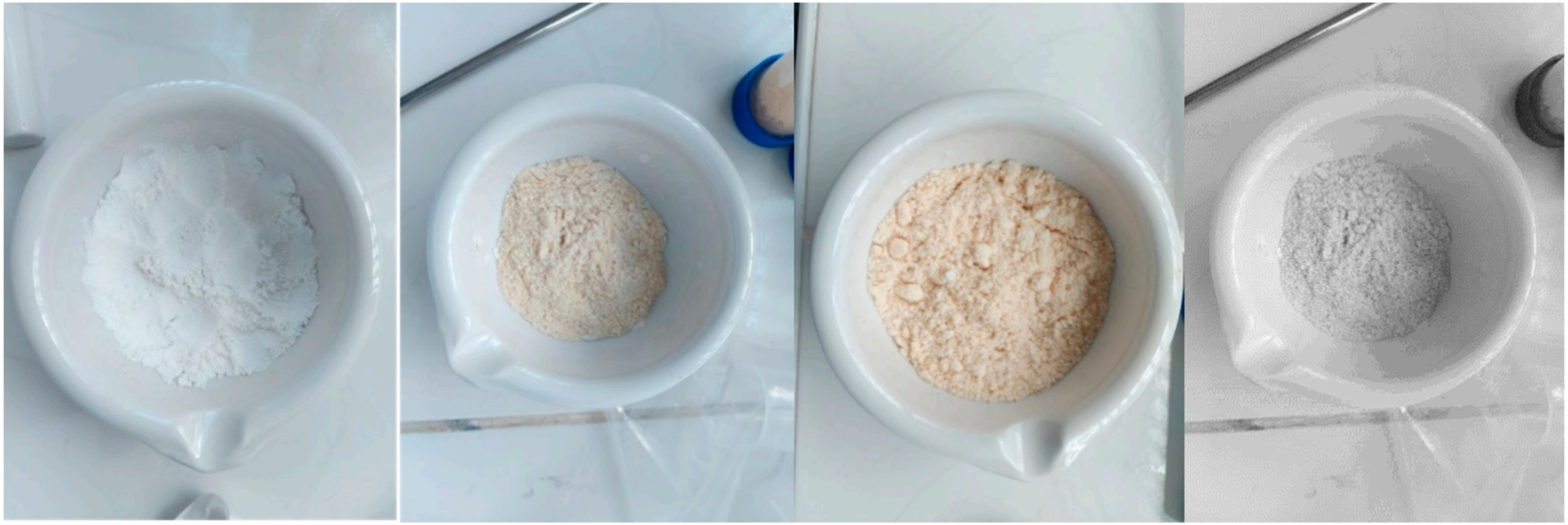

(a) (b) (c) (d)

Figure S1. Citrus peel essential oils nanoencapsulated with different types of wall material

Figure S1shows the particles obtained after the lyophilization process. The essential oils nanoencapsulated with GA/MD and AG/CAS were whitish (Figure 1 a and d). In contrast, the essential oils nanoencapsulated with MD/CAS and AG/MD/CAS were yellowish due to the casein used for the wall material (Figures 1 b and c).

Table S1. Values of physicochemical indicators evaluated in essential oil nanoencapsulates.

Sample	Nanoencapsulated	Yield	MC	Solubility	Hygroscopicity	TPC	DPPH	ABTS	Efficiency	DLimonene
C. limetta sp.	GA/MD	89.94	6.80	94.00	6.00	159.40	78.05	1598.00	76.59	614.76
C. limetta sp.	GA/MD	89.94	6.80	86.00	6.00	167.90	80.49	1645.50	75.34	629.57
C. limetta sp.	GA/MD	89.94	6.80	90.00	6.00	164.90	80.10	1645.50	75.78	593.87
C. limetta sp.	GA/MD	89.94	6.80	90.00	6.00	175.40	84.34	1685.50	74.24	613.39
C. limetta sp.	GA/CAS	94.24	6.07	46.00	8.00	221.40	69.70	1520.50	67.48	497.63
C. limetta sp.	GA/CAS	94.24	6.07	50.00	10.00	236.40	69.45	1553.00	65.28	552.31
C. limetta sp.	GA/CAS	94.24	6.07	48.00	8.00	231.90	66.75	1555.50	65.94	563.78
C. limetta sp.	GA/CAS	94.24	6.07	48.00	8.00	223.40	70.60	1518.00	67.18	574.12
C. limetta sp.	MD/CAS	96.41	5.04	48.00	16.00	169.40	66.50	1350.50	75.12	562.91
C. limetta sp.	MD/CAS	96.41	5.04	48.00	18.00	172.40	68.55	1360.50	74.68	543.46
C. limetta sp.	MD/CAS	96.41	5.04	48.00	16.00	166.90	66.37	1345.50	75.48	550.07
C. limetta sp.	MD/CAS	96.41	5.04	48.00	16.00	164.40	67.27	1350.50	75.85	571.06
C. limetta sp.	GA/MD/CAS	83.71	4.57	62.00	14.00	198.90	79.46	1685.50	70.78	317.61
C. limetta sp.	GA/MD/CAS	83.71	4.57	62.00	12.00	205.90	78.95	1725.50	69.76	325.63
C. limetta sp.	GA/MD/CAS	83.71	4.57	64.00	12.00	205.40	80.23	1720.50	69.83	307.66
C. limetta sp.	GA/MD/CAS	83.71	4.57	64.00	12.00	210.90	78.43	1703.00	69.02	341.89
C. reticulata	GA/MD	95.75	6.06	88.00	8.00	48.40	68.94	1220.50	88.48	337.62
C. reticulata	GA/MD	95.75	6.06	90.00	8.00	160.40	71.53	1298.00	61.83	318.32
C. reticulata	GA/MD	95.75	6.06	94.00	6.00	157.40	69.80	1215.50	62.55	312.50
C. reticulata	GA/MD	95.75	6.06	94.00	6.00	157.40	67.57	1288.00	62.55	314.48
C. reticulata	GA/CAS	90.29	7.36	82.00	8.00	146.90	66.96	1475.50	65.05	415.10
C. reticulata	GA/CAS	90.29	7.36	76.00	8.00	149.90	70.54	1503.00	64.33	413.44
C. reticulata	GA/CAS	90.29	7.36	78.00	8.00	142.90	70.30	1520.50	66.00	422.11
C. reticulata	GA/CAS	90.29	7.36	78.00	8.00	144.90	68.32	1535.50	65.52	394.50
C. reticulata	MD/CAS	69.25	5.01	76.00	16.00	147.40	69.80	1463.00	64.93	731.28
C. reticulata	MD/CAS	69.25	5.01	78.00	16.00	142.90	72.77	1555.50	66.00	729.44

C. reticulata	MD/CAS	69.25	5.01	72.00	16.00	143.40	71.78	1555.50	65.88	694.85
C. reticulata	MD/CAS	69.25	5.01	72.00	16.00	148.40	71.04	1475.50	64.69	726.78
C. reticulata	GA/MD/CAS	93.08	6.14	60.00	8.00	148.90	70.42	1595.50	64.57	394.15
C. reticulata	GA/MD/CAS	93.08	6.14	58.00	10.00	147.90	73.76	1615.50	64.81	377.50
C. reticulata	GA/MD/CAS	93.08	6.14	60.00	8.00	156.40	73.27	1588.00	62.79	388.46
C. reticulata	GA/MD/CAS	93.08	6.14	60.00	8.00	147.40	70.30	1598.00	64.93	369.47
C. limetta Risso	GA/MD	90.86	5.68	98.00	6.00	70.40	68.53	1083.00	67.67	142.62
C. limetta Risso	GA/MD	90.86	5.68	76.00	8.00	70.40	71.45	1103.00	67.67	142.56
C. limetta Risso	GA/MD	90.86	5.68	72.00	8.00	66.40	71.57	1080.50	69.51	142.57
C. limetta Risso	GA/MD	90.86	5.68	72.00	8.00	67.40	68.89	1093.00	69.05	133.08
C. limetta Risso	GA/CAS	92.67	7.34	46.00	10.00	95.90	67.07	1178.00	55.96	539.82
C. limetta Risso	GA/CAS	92.67	7.34	44.00	10.00	89.90	69.38	1193.00	58.72	535.85
C. limetta Risso	GA/CAS	92.67	7.34	42.00	12.00	91.90	69.50	1203.00	57.80	520.72
C. limetta Risso	GA/CAS	92.67	7.34	42.00	12.00	97.40	66.46	1218.00	55.28	548.02
C. limetta Risso	MD/CAS	96.62	4.23	52.00	8.00	96.90	68.17	1215.50	55.51	263.27
C. limetta Risso	MD/CAS	96.62	4.23	42.00	6.00	96.40	71.08	1225.50	55.74	254.85
C. limetta Risso	MD/CAS	96.62	4.23	44.00	6.00	102.90	71.45	1213.00	52.75	252.49
C. limetta Risso	MD/CAS	96.62	4.23	44.00	6.00	97.40	68.17	1223.00	55.28	267.13
C. limetta Risso	GA/MD/CAS	95.00	5.11	56.00	8.00	92.90	71.08	1095.50	57.34	376.70
C. limetta Risso	GA/MD/CAS	95.00	5.11	66.00	8.00	87.40	68.17	1123.00	59.87	379.41
C. limetta Risso	GA/MD/CAS	95.00	5.11	64.00	6.00	95.90	68.77	1115.50	55.96	381.81
C. limetta Risso	GA/MD/CAS	95.00	5.11	64.00	6.00	90.90	66.71	1060.50	58.26	408.44
C. sinensis	GA/MD	92.76	5.07	94.00	10.00	58.40	70.69	1040.50	48.22	211.27
C. sinensis	GA/MD	92.76	5.07	92.00	10.00	67.90	69.15	978.00	39.79	226.77
C. sinensis	GA/MD	92.76	5.07	94.00	10.00	68.90	70.69	1015.50	38.91	217.68
C. sinensis	GA/MD	92.76	5.07	94.00	10.00	66.90	67.61	1013.00	40.68	217.32
C. sinensis	GA/CAS	86.59	4.55	54.00	10.00	76.40	67.61	1045.50	32.26	294.46
C. sinensis	GA/CAS	86.59	4.55	52.00	10.00	80.90	68.44	1058.00	28.27	294.59
C. sinensis	GA/CAS	86.59	4.55	52.00	10.00	88.40	68.32	1055.50	21.62	280.74
C. sinensis	GA/CAS	86.59	4.55	52.00	10.00	88.40	67.61	1078.00	21.62	284.83
C. sinensis	MD/CAS	96.06	4.78	52.00	8.00	82.90	68.68	1008.00	26.49	412.34
C. sinensis	MD/CAS	96.06	4.78	52.00	8.00	88.40	70.21	1035.50	21.62	402.88

C. sinensis	MD/CAS	96.06	4.78	52.00	8.00	85.90	69.15	1025.50	23.83	440.31
C. sinensis	MD/CAS	96.06	4.78	52.00	8.00	85.90	65.72	973.00	23.83	400.60
C. sinensis	GA/MD/CAS	87.41	6.24	62.00	6.00	76.40	65.13	1013.00	32.26	578.98
C. sinensis	GA/MD/CAS	87.41	6.24	62.00	6.00	80.90	66.19	1035.50	28.27	565.80
C. sinensis	GA/MD/CAS	87.41	6.24	60.00	8.00	79.40	66.67	1028.00	29.60	497.63
C. sinensis	GA/MD/CAS	87.41	6.24	60.00	8.00	81.40	68.09	1020.50	27.82	442.10

Table S2. Retention Time (RT), Retention Index (RI), and Linear Retention Index (LRI) of Volatile Compounds Identified by GC-MS.

EO	MP	Compounds	RT	RI	LRI
C. limetta sp.	GA/MD	D-Limonene	26.91	1041	1018
C. limetta sp.	GA/MD	6-Octen-1-ol, 7-methyl-3-methylene-	33.01	1157	1195
C. limetta sp.	GA/MD	Citronellol	36.65	1229	1228
C. limetta sp.	GA/MD	Thymol	39.88	1292	1291
C. limetta sp.	GA/CAS	D-Limonene	26.91	1041	1018
C. limetta sp.	GA/CAS	Citronellol	36.65	1229	1228
C. limetta sp.	GA/CAS	Thymol	39.89	1292	1291
C. limetta sp.	MD/CAS	D-Limonene	26.91	1041	1018
C. limetta sp.	MD/CAS	6-Octen-1-ol, 3,7-dimethyl-, (R)-	36.65	1229	1220
C. limetta sp.	MD/CAS	Thymol	39.89	1292	1291
C. limetta sp.	GA/MD/CAS	D-Limonene	26.90	1041	1018
C. limetta sp.	GA/MD/CAS	Thymol	39.89	1292	1291
C. reticulata	GA/MD	2-Butenoic acid, methyl ester, (Z)-	9.17	706	694
C. reticulata	GA/MD	Hexane, 2,5-dimethyl-	10.97	741	729
C. reticulata	GA/MD	D-Limonene	26.90	1041	1018
C. reticulata	GA/MD	.gamma.-Terpinene	28.37	1068	1060
C. reticulata	GA/MD	Thymol	39.89	1292	1291
C. reticulata	GA/CAS	Oxetane, 2,2,4-trimethyl-	9.55	713	664
C. reticulata	GA/CAS	Butane, 1-ethoxy-	9.55	713	669
C. reticulata	GA/CAS	Hexane, 2,5-dimethyl-	10.97	741	729
C. reticulata	GA/CAS	D-Limonene	26.91	1041	1018
C. reticulata	GA/CAS	.gamma.-Terpinene	28.37	1068	1060
C. reticulata	GA/CAS	Thymol	39.89	1292	1291

C. reticulata	GA/CAS	Benzoic acid, 2-(methylamino)-, methyl ester	46.23	1435	1408
C. reticulata	GA/CAS	2,6,9,11-Dodecatetraenal, 2,6,10-trimethyl-, (E,E,E)-	59.80	1765	1752
C. reticulata	MD/CAS	Butane, 1-ethoxy-	9.54	713	669
C. reticulata	MD/CAS	Hexane, 2,5-dimethyl-	10.97	741	729
C. reticulata	MD/CAS	D-Limonene	26.91	1041	1018
C. reticulata	MD/CAS	.gamma.-Terpinene	28.37	1068	1060
C. reticulata	MD/CAS	Thymol	39.89	1292	1291
C. reticulata	GA/MD/CAS	Formic acid, 1,1-dimethylethyl ester	9.54	713	587
C. reticulata	GA/MD/CAS	Hexane, 2,5-dimethyl-	10.97	741	729
C. reticulata	GA/MD/CAS	D-Limonene	26.90	1041	1018
C. reticulata	GA/MD/CAS	.gamma.-Terpinene	28.37	1068	1060
C. reticulata	GA/MD/CAS	Thymol	39.89	1292	1291
C. limetta Risso	GA/MD	D-Limonene	26.90	1041	1018
C. limetta Risso	GA/MD	trans-Ocimenol	35.84	1041	1018
C. limetta Risso	GA/MD	1,6-Octadien-3-ol, 3,7-dimethyl-, formate	37.78	1251	1215
C. limetta Risso	GA/CAS	D-Limonene	26.90	1041	1018
C. limetta Risso	GA/CAS	Linalool	30.20	1102	1099
C. limetta Risso	GA/CAS	.alpha.-Terpineol	35.83	1212	1189
C. limetta Risso	GA/CAS	Linalyl acetate	37.78	1251	1257
C. limetta Risso	GA/CAS	2,6-Octadien-1-ol, 3,7-dimethyl-, (Z)-	37.90	1253	1228
C. limetta Risso	MD/CAS	D-Limonene	26.90	1041	1018
C. limetta Risso	MD/CAS	1,6-Octadien-3-ol, 3,7-dimethyl-, formate	37.77	1251	1215
C. limetta Risso	GA/MD/CAS	D-Limonene	26.90	1041	1018
C. limetta Risso	GA/MD/CAS	Linalool	30.20	1102	1099
C. limetta Risso	GA/MD/CAS	.alpha.-Terpineol	35.83	1213	1189
C. limetta Risso	GA/MD/CAS	Thymol	39.89	1292	1291
C. sinensis	GA/MD	Oxetane, 2,2,4-trimethyl-	9.54	713	664
C. sinensis	GA/MD	D-Limonene	26.90	1041	1018
C. sinensis	GA/CAS	Oxetane, 2,2,4-trimethyl-	9.54	713	664
C. sinensis	GA/CAS	D-Limonene	26.90	1041	1018
C. sinensis	MD/CAS	Oxetane, 2,2,4-trimethyl-	9.54	713	664
C. sinensis	MD/CAS	D-Limonene	26.90	1041	1018

C. sinensis	GA/MD/CAS	Oxetane, 2,2,4-trimethyl-	9.54	713	664
C. sinensis	GA/MD/CAS	D-Limonene	26.90	1041	1018
C. sinensis	GA/MD/CAS	1-Octanol	28.36	1068	1071
C. sinensis	GA/MD/CAS	Linalool	30.19	1102	1099
C. sinensis	GA/MD/CAS	Butylated Hydroxytoluene	50.02	1521	1513

Table S3. Concentration of D-limonene in citrus peel essential oils nanoencapsulated with different types of wall material.

Sample	Wall	D-Limonene (ng/ml) sample	Dilution factor	D-Limonene (ng/ml)
C. limetta sp.	GA/MD	61.4759	10	614.759
C. limetta sp.	GA/MD	62.9565	10	629.565
C. limetta sp.	GA/MD	59.3868	10	593.868
C. limetta sp.	GA/MD	61.3390	10	613.39
C. limetta sp.	GA/CAS	49.7633	10	497.633
C. limetta sp.	GA/CAS	55.2306	10	552.306
C. limetta sp.	GA/CAS	56.3780	10	563.78
C. limetta sp.	GA/CAS	57.4115	10	574.115
C. limetta sp.	MD/CAS	56.2910	10	562.91
C. limetta sp.	MD/CAS	54.3463	10	543.463
C. limetta sp.	MD/CAS	55.0073	10	550.073
C. limetta sp.	MD/CAS	57.1060	10	571.06
C. limetta sp.	MD/GA/CAS	31.7611	10	317.611
C. limetta sp.	MD/GA/CAS	32.5625	10	325.625
C. limetta sp.	MD/GA/CAS	30.7656	10	307.656
C. limetta sp.	MD/GA/CAS	34.1891	10	341.891
C. reticulata	MD/GA	33.7623	10	337.623
C. reticulata	MD/GA	31.8320	10	318.32
C. reticulata	MD/GA	31.2501	10	312.501
C. reticulata	MD/GA	31.4477	10	314.477
C. reticulata	GA/CAS	41.5101	10	415.101
C. reticulata	GA/CAS	41.3436	10	413.436

C. reticulata	GA/CAS	42.2112	10	422.112
C. reticulata	GA/CAS	39.4496	10	394.496
C. reticulata	MD/CAS	73.1279	10	731.279
C. reticulata	MD/CAS	72.9443	10	729.443
C. reticulata	MD/CAS	69.4849	10	694.849
C. reticulata	MD/CAS	72.6781	10	726.781
C. reticulata	GA/MD/CAS	39.4146	10	394.146
C. reticulata	GA/MD/CAS	37.7498	10	377.498
C. reticulata	GA/MD/CAS	38.8456	10	388.456
C. reticulata	GA/MD/CAS	36.9472	10	369.472
C. limetta Risso	GA/MD	14.2619	10	142.619
C. limetta Risso	GA/MD	14.2562	10	142.562
C. limetta Risso	GA/MD	14.2570	10	142.57
C. limetta Risso	GA/MD	13.3080	10	133.08
C. limetta Risso	GA/CAS	53.9818	10	539.818
C. limetta Risso	GA/CAS	53.5851	10	535.851
C. limetta Risso	GA/CAS	52.0723	10	520.723
C. limetta Risso	GA/CAS	54.8020	10	548.02
C. limetta Risso	MD/CAS	26.3270	10	263.27
C. limetta Risso	MD/CAS	25.4849	10	254.849
C. limetta Risso	MD/CAS	25.2490	10	252.49
C. limetta Risso	MD/CAS	26.7125	10	267.125
C. limetta Risso	MD/GA/CAS	37.6704	10	376.704
C. limetta Risso	MD/GA/CAS	37.9407	10	379.407
C. limetta Risso	MD/GA/CAS	38.1808	10	381.808
C. limetta Risso	MD/GA/CAS	40.8440	10	408.44
C. sinensis	GA/MD	21.1272	10	211.272
C. sinensis	GA/MD	22.6767	10	226.767
C. sinensis	GA/MD	21.7684	10	217.684

C. sinensis	GA/MD	21.7317	10	217.317
C. sinensis	GA/CAS	29.4456	10	294.456
C. sinensis	GA/CAS	29.4586	10	294.586
C. sinensis	GA/CAS	28.0736	10	280.736
C. sinensis	GA/CAS	28.4832	10	284.832
C. sinensis	MD/CAS	41.2340	10	412.34
C. sinensis	MD/CAS	40.2879	10	402.879
C. sinensis	MD/CAS	44.0309	10	440.309
C. sinensis	MD/CAS	40.0601	10	400.601
C. sinensis	MD/GA/CAS	38.5985	15	578.9775
C. sinensis	MD/GA/CAS	37.7201	15	565.8015
C. sinensis	MD/GA/CAS	33.1754	15	497.631
C. sinensis	MD/GA/CAS	29.4731	15	442.0965

Table S4. Volatile compounds of citrus peel essential oils nanoencapsulated with different types of wall material.

**Sample**	**Nanoencapsulated**	**Compuesto**	**Abundancia**
C. limetta sp.	GA/MD	D-Limonene	80.45
C. limetta sp.	GA/MD	Thymol	5.96
C. limetta sp.	GA/MD	Citronellol	9.66
C. limetta sp.	GA/MD	6-Octen-1-ol, 7-methyl-3-methylene-	3.94
C. limetta sp.	GA/CAS	D-Limonene	65.15
C. limetta sp.	GA/CAS	Thymol	8.48
C. limetta sp.	GA/CAS	Citronellol	26.38
C. limetta sp.	MD/CAS	D-Limonene	90.65
C. limetta sp.	MD/CAS	6-Octen-1-ol, 3,7-dimethyl-, (R)-	3.14
C. limetta sp.	MD/CAS	Thymol	6.21
C. limetta sp.	GA/MD/CAS	D-Limonene	89.21
C. limetta sp.	GA/MD/CAS	Thymol	10.79
C. reticulata	GA/MD	D-Limonene	54.89
C. reticulata	GA/MD	2-Butenoic acid, methyl ester, (Z)-	3.50
C. reticulata	GA/MD	Hexane, 2,5-dimethyl-	8.07

C. reticulata	GA/MD	.gamma.-Terpinene	10.85
C. reticulata	GA/MD	Thymol	22.69
C. reticulata	GA/CAS	Oxetane, 2,2,4-trimethyl-	6.06
C. reticulata	GA/CAS	Butane, 1-ethoxy-	6.50
C. reticulata	GA/CAS	Benzoic acid, 2-(methylamino)-, methyl ester	4.24
C. reticulata	GA/CAS	D-Limonene	43.45
C. reticulata	GA/CAS	2,6,9,11-Dodecatetraenal, 2,6,10-trimethyl-, (E,E,E)-	9.64
C. reticulata	GA/CAS	Hexane, 2,5-dimethyl-	5.12
C. reticulata	GA/CAS	.gamma.-Terpinene	7.87
C. reticulata	GA/CAS	Thymol	17.12
C. reticulata	MD/CAS	Butane, 1-ethoxy-	5.68
C. reticulata	MD/CAS	D-Limonene	65.28
C. reticulata	MD/CAS	Hexane, 2,5-dimethyl-	4.28
C. reticulata	MD/CAS	.gamma.-Terpinene	12.76
C. reticulata	MD/CAS	Thymol	12.00
C. reticulata	GA/MD/CAS	Formic acid, 1,1-dimethylethyl ester	7.26
C. reticulata	GA/MD/CAS	D-Limonene	54.99
C. reticulata	GA/MD/CAS	Hexane, 2,5-dimethyl-	6.52
C. reticulata	GA/MD/CAS	.gamma.-Terpinene	9.89
C. reticulata	GA/MD/CAS	Thymol	21.33
C. limetta Risso	GA/MD	1,6-Octadien-3-ol, 3,7-dimethyl-, formate	36.99
C. limetta Risso	GA/MD	D-Limonene	52.30
C. limetta Risso	GA/MD	trans-Ocimenol	10.71
C. limetta Risso	GA/CAS	2,6-Octadien-1-ol, 3,7-dimethyl-, (Z)-	9.82
C. limetta Risso	GA/CAS	.alpha.-Terpineol	4.47
C. limetta Risso	GA/CAS	D-Limonene	43.36
C. limetta Risso	GA/CAS	Linalool	20.77
C. limetta Risso	GA/CAS	Linalyl acetate	21.58
C. limetta Risso	MD/CAS	1,6-Octadien-3-ol, 3,7-dimethyl-, formate	19.54
C. limetta Risso	MD/CAS	D-Limonene	80.46
C. limetta Risso	GA/MD/CAS	.alpha.-Terpineol	5.42
C. limetta Risso	GA/MD/CAS	D-Limonene	66.47

C. limetta Risso	GA/MD/CAS	Linalool	19.36
C. limetta Risso	GA/MD/CAS	Thymol	8.75
C. sinensis	GA/MD	Oxetane, 2,2,4-trimethyl-	18.70
C. sinensis	GA/MD	D-Limonene	81.30
C. sinensis	GA/CAS	Oxetane, 2,2,4-trimethyl-	14.13
C. sinensis	GA/CAS	D-Limonene	85.87
C. sinensis	MD/CAS	Oxetane, 2,2,4-trimethyl-	9.75
C. sinensis	MD/CAS	D-Limonene	90.25
C. sinensis	GA/MD/CAS	Oxetane, 2,2,4-trimethyl-	6.03
C. sinensis	GA/MD/CAS	D-Limonene	51.51
C. sinensis	GA/MD/CAS	Butylated Hydroxytoluene	11.23
C. sinensis	GA/MD/CAS	Linalool	27.44
C. sinensis	GA/MD/CAS	1-Octanol	3.79
